# Prevalence and Correlates of Depression Among Patients With Drug-Susceptible Tuberculosis Enrollees in Ogbomoso, Oyo State: A Cross-Sectional Study

**DOI:** 10.7759/cureus.100472

**Published:** 2025-12-31

**Authors:** Sunday Olarewaju, Sunday C Adeyemo, Ayodele Ajayi, Obehi F Are-Daniel, Anu Chayelil Santhosh, Eniola D Olabode, Ayomide Timilehin, Janet O Ayinmodu, Olushina O Oladeji, John T Odedele, Doyin V Olaniyan, Oluwatoba J Oyedeji, Zainab A Abdulsalam

**Affiliations:** 1 Community Medicine, Osun State University, Osogbo, NGA; 2 Public Health Research, University of Wolverhampton, Wolverhampton, GBR; 3 Health and Biomedical Sciences, Institut Superieur de Sante, Niamey, NER; 4 Nursing, Pear Tree Court Care Home - Care UK, Waterlooville, GBR; 5 Psychiatry, Afe Babalola University Teaching Hospital, Ado-Ekiti, NGA; 6 Acute Mental Health, Herefordshire and Worcestershire Health and Care NHS Trust, Hereford, GBR; 7 Health Sciences, Hertford Regional College, Hertfordshire, GBR; 8 Community Medicine, Institut Superieur de Sante, Niamey, NGA; 9 Medicine and Surgery, Ladoke Akintola University of Technology, Ogbomosho, NGA; 10 College of Health Sciences, Ladoke Akintola University of Technology, Ogbomosho, NGA

**Keywords:** depression, directly observed therapy, drug-susceptible, ogbomosho, tuberculosis

## Abstract

Background: Tuberculosis is an infectious disease that continues to present a major public health challenge. There is a recognized correlation between the condition and depression. This study aims to find out the prevalence and pattern of depression among drug-susceptible TB patients to improve treatment outcomes.

Methodology: The study was a cross-sectional hospital-based survey across the directly observed therapy (DOT) centers in Ogbomosho. Sample size of 333 respondents was calculated using Leslie Fischer's formula (n = z²pq/d²). A multistage sampling technique was used to select respondents. Data was collected using a pre-tested semistructured questionnaire and analyzed using Statistical Package for Social Sciences (SPSS), version 20 (IBM Corp., Armonk, NY). The questionnaire covered sociodemographic characteristics, lifestyle, and comorbidities and included the Patient Health Questionnaire-9 (PHQ-9) to assess depression. Respondents who scored 0-4 were categorized as having no depression, those who scored 5-9 were categorized as having mild depression, those who scored 10-14 were categorized as having moderate depression, while those who scored 15-19 were categorized as having moderately severe depression. Descriptive analysis was done on all variables. Bivariate and multivariate analyses were done using chi-square and binary logistic regression, respectively. The level of significance is set with a p-value less than 0.05.

Results: The overall prevalence of depression was 146 (43.8%). Among those who were depressed, 96 (65.6%) had mild depression, 36 (24.7%) had moderate depression, while 14 (9.7%) had moderately severe depression. Sex, marital status, level of education, and average monthly income were significantly associated with depression status at the bivariate level. Multivariate analysis revealed that level of education (AOR = 0.175, P = 0.001) and living with HIV (AOR = 35.303, P = 0.017) were significantly associated with depression status.

Conclusion: This study found a high prevalence of depression among TB patients. Factors such as level of education and comorbidities like TB/HIV and diabetes mellitus were statistically associated with depression.

## Introduction

Tuberculosis (TB), an infectious disease caused by *Mycobacterium tuberculosis*, is still one of the leading public health problems despite advances in efforts to reduce its incidence, morbidity, and mortality [1]. In 2021, the WHO reported that about 10.6 million people were infected with tuberculosis, with an estimated 23% of the global burden and 33% of global TB deaths occurring in Africa [2]. Nigeria ranks first in Africa and is one of the 10 countries with the highest number of missing TB cases. It ranks sixth globally, contributing almost 4.6% of the global burden [3]. Nigeria also bears a high triple burden of drug-susceptible TB, a bacteriologically confirmed or clinically diagnosed case of TB without evidence of resistance to rifampicin and isoniazid [4,5].

Depression is a mental state characterized by loss of interest, feelings of guilt, disturbed sleep or appetite, loss of self-worth, and usually suicidal thoughts [1,2]. Depression is a common mental disorder, with about 5% of adults affected globally. It is a major cause of suicide, with more than 700,000 people dying by suicide every year [6]. Chronic pain, frequent hospital admissions, and dependency on the hospital, which is common among TB patients, have been reported to be associated with depression [7,8]. Studies have shown that the prevalence of depression correlates with the severity and duration of tuberculosis [2,7]. Depression in TB patients is often due to the nature of the infection, side effects of medications, and other social determinants of health. Several studies have shown a higher prevalence of depression among TB patients compared to the general population. A study conducted in Nigeria by Ige and Lasebikan reported a prevalence of about 45.5% [5].

When TB and depression coexist, patients tend to suffer in silence, and when poor medication compliance accompanies this, mortality rates increase. Previous studies have identified a low degree of suspicion of depression among clinicians managing TB patients [4,5,7]. Therefore, addressing the psychosocial issues faced by patients undergoing TB treatment and improving consultation-liaison psychiatric services may optimize adherence and enhance treatment success. It is therefore important to assess the prevalence and pattern of depression among drug-susceptible TB patients to improve treatment outcomes and reduce morbidity and mortality from the disease. Hence, this study aims to assess the prevalence and pattern of depression, as well as factors associated with depression among drug-susceptible TB patients.

## Materials and methods

Study design and setting

This study was carried out in Ogbomoso, Southwestern Nigeria. It was a cross-sectional, hospital-based survey employing a quantitative data collection method (use of a questionnaire). The study was conducted across the directly observed therapy (DOT) centers in selected local governments in Ogbomoso. DOT centers owned by both state and local governments were included in the study.

Study population and eligibility criteria

TB patients aged 18 years and above, resident in Ogbomosho, who had been on anti-TB drugs for at least two months, had confirmed drug susceptibility, and who were mentally capable of providing consent were included. Exclusion criteria were newly diagnosed and unregistered pulmonary TB patients, pregnant women with pulmonary TB, severely ill or debilitated patients, patients with extra-pulmonary TB, and those unable to provide consent. This was to avoid cognitive bias. A written consent form was signed by each respondent.

Sample size determination

The sample size was calculated using Leslie Fischer's formula: $n = \frac{z^{2}pq}{d^{2}}$ [3], applying a previously reported prevalence of depression among drug-susceptible TB patients (27% or 0.27) [2]. With an additional 10% allowance for non-response, a total of 333 questionnaires were administered.

Sampling technique

A multistage sampling technique was used:

First Stage

From the list of urban and rural local government areas in Ogbomoso, Ogbomoso North and Ogooluwa local government areas were selected.

Second Stage

A list of all registered DOT centers in Ogbomoso was obtained from the Tuberculosis and Leprosy Supervisor for each local government area. All DOT centers were included.

Third Stage

Proportional allocation was used to determine the number of clients to be selected from each DOT center. A systematic sampling method was used to select clients from each center. Sampling interval (k) was calculated by dividing the number of TB patients in the center by the number of clients to be selected from the center. The first respondent was selected randomly, while subsequent respondents were selected using the kth number until the target sample was reached.

Data collection

Data collection took place from January 21 to January 31, 2025, using a semistructured questionnaire, which was pre-tested among TB patients in the DOT center in Ogbomoso South local government area. The internal consistency of the questionnaire was calculated, and the questionnaire was adjusted based on responses from the pre-test. It was then interviewer-administered by medical students in their final year who had been trained in data collection under the supervision of the lead researcher, who is a doctor. The questionnaire covered sociodemographic characteristics, lifestyle, and comorbidities (which were self-reported) and included the Patient Health Questionnaire-9 (PHQ-9) [9], which is an open-access questionnaire administered in English to assess depression. Respondents who scored 0-4 were categorized as having no depression, those who scored 5-9 were categorized as having mild depression, those who scored 10-14 were categorized as having moderate depression, those who scored 15-19 were categorized as having moderately severe depression, while those who scored 20-27 were categorized as having severe depression.

Statistical analysis

At the end of data collection, questionnaires were reviewed for errors and omissions and corrected before leaving each respondent. Data were then entered into the computer and analyzed using SPSS version 20 (IBM Corp., Armonk, NY). Descriptive statistics were conducted for all variables. Bivariate and multivariate analyses were performed using Chi-square tests and binary logistic regression, respectively. A p-value of less than 0.05 was considered statistically significant.

## Results

Sociodemographic characteristics of respondents

The sociodemographic characteristics of the respondents revealed that the majority, 109 (32.7%), were within the age range of 30-40 years. Most respondents, 216 (64.9%), were married. Regarding education, the largest proportion, 108 (32.4%), had a tertiary education. The predominant ethnic group was Yoruba, 271 (81.4%). Most respondents, 174 (52.3%), were Christians (Table 1).

**Table 1:** Association between sociodemographic status and depression status Data has been presented in frequency and percentage. * indicates significance. The empty cells are not applicable. χ^2^: Chi-square; df: Degree of freedom; p: Probability value.

Variables	Not Depressed (n = 187)	Depressed (n = 146)	χ²	df	p
Age (Years)			1.23	4	0.873
<30	23 (12.3%)	14 (9.6%)			
30–40	55 (29.4%)	54 (37.0%)			
41–50	47 (25.1%)	33 (22.6%)			
51–60	39 (20.9%)	26 (17.8%)			
>60	23 (12.3%)	19 (13.0%)			
Sex			6.643	1	0.010*
Male	110 (58.8%)	55 (37.7%)			
Female	77 (41.2%)	91 (62.3%)			
Religion			0.581	2	0.748
Christian	92 (52.2%)	82 (56.2%)			
Muslim	88 (44.9%)	59 (40.4%)			
Traditional	7 (2.9%)	5 (3.4%)			
Marital Status			23.948	3	<0.001*
Single	34 (18.2%)	43 (29.5%)			
Married	138 (73.8%)	78 (53.4%)			
Widow	9 (4.8%)	19 (13.0%)			
Widower	7 (3.7%)	6 (4.1%)			
Ethnicity			6.904	3	0.075
Yoruba	156 (81.6%)	115 (78.8%)			
Igbo	6 (2.9%)	15 (10.3%)			
Hausa	20 (11.8%)	13 (8.9%)			
Others	5 (3.7%)	3 (2.1%)			
Level of Education			11.81	3	0.008*
No formal education	21 (11.2%)	38 (26.1%)			
Primary	42 (22.5%)	45 (30.8%)			
Secondary	46 (24.6%)	33 (22.6%)			
Tertiary	78 (41.7%)	30 (20.5%)			
Occupational Status			3.498	2	0.174
Employed	124 (66.3%)	91 (62.3%)			
Unemployed	54 (28.9%)	46 (31.5%)			
Student	9 (4.8%)	9 (6.2%)			
Average Monthly Income			12.71	1	<0.001*
< ₦30,000	59 (31.6%)	71 (48.6%)			
≥ ₦30,000	128 (68.4%)	75 (51.4%)			

Assessment of depression among respondents

Over half of the participants (50.5%) reported no signs of depression, such as a lack of interest or feeling hopeless. Most did not feel bad about themselves (56.8%) nor experienced slowed movements or restlessness (69.7%). A majority (67.6%) had no thoughts of self-harm. However, common symptoms included trouble sleeping (51.4%) and poor appetite or overeating (46.2%), both reported on "several days." Concentration problems were mostly absent in 46.2% of respondents.

**Figure 1:**
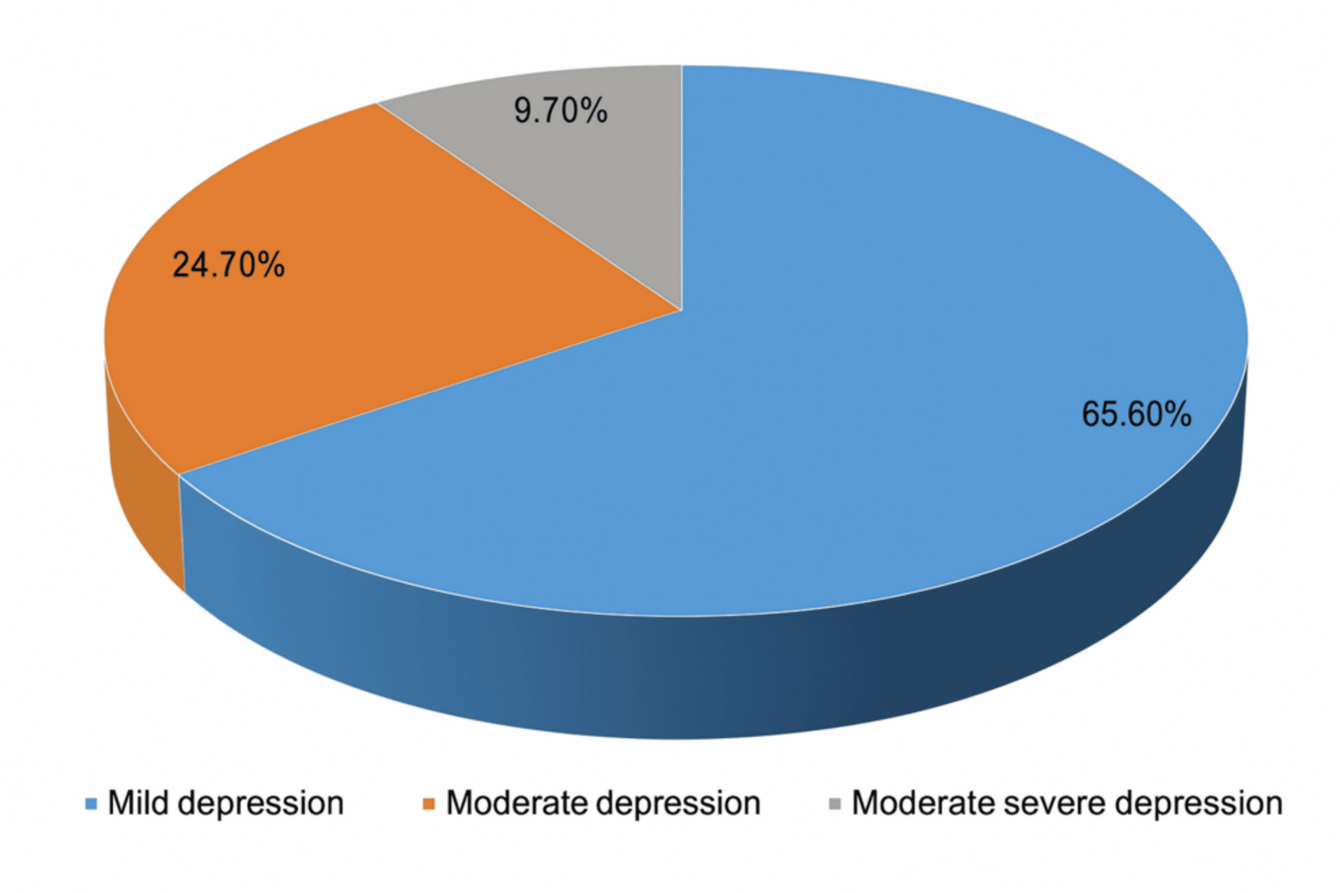
Categories of depression The data has been presented in frequency and percentage.

The overall prevalence of depression was 146 (43.8%). Among those who were depressed, 96 (65.6%) had mild depression, 36 (24.7%) had moderate depression, while 14 (9.7%) had moderately severe depression (Figure 1).

Association of HIV status and self-reported hypertension and diabetes status with depression

Results revealed that HIV status (χ² = 61.381, P < 0.001) and diabetes (χ² = 7.295, P = 0.026) were significantly associated with depression status among drug-susceptible tuberculosis patient enrollees in Ogbomoso, Oyo state (Table 2).

**Table 2:** Association of HIV status, hypertension, and diabetes with depression * Significant at P < 0.050. χ^2^: Pearson Chi-square value; df: Degree of freedom; P: Probability value.

Variables	Categories	Depression		χ^2^	df	P-value
		Not Depressed (n = 187)	Depressed (n = 146)			
HIV status	Positive	20 (10.7)	60 (41.1)	61.381	1	<0.001*
	Negative	167 (89.3)	86 (58.9)			
Diabetes	Yes	19 (10.2)	30 (16.5)	7.295	1	0.026*
	No	168 (89.8)	116 (79.5)			
Hypertension	Yes	37 (19.8)	36 (24.7)	4.446	1	0.108
	No	150 (80.2)	110 (75.3)			

Predictors of depression status among drug-susceptible tuberculosis patient enrollees in Ogbomoso, Oyo State

Results revealed that respondents with no formal education had lower odds of depression when compared to the reference category (AOR = 0.175, P = 0.001). Respondents with a primary level of education were two times less likely to develop depression (AOR = 0.427, P = 0.023). Respondents with a secondary level of education (AOR = 0.374, P = 0.020) were three times less likely to develop depression compared to a tertiary level of education. Respondents living with HIV were 35 times more likely to develop depression (AOR = 35.303, P = 0.017) compared to those who were HIV negative (Table 3).

**Table 3:** Predictors of depression status among drug-susceptible tuberculosis patient enrollees in Ogbomoso, Oyo State * indicates significance. AOR: Adjusted Odds ratio; CI: Confidence interval; P: Probability value; R: Reference. Empty spaces indicate values that are not applicable.

Variables	AOR	95% CI (Lower)	95% CI (Upper)	p-value
Sex				
Male	0.669	0.363	1.234	0.198
Female (Ref)	—	—	—	—
Marital Status				
Single	1.606	0.283	9.111	0.593
Married	1.071	0.221	5.179	0.932
Widow	3.836	0.585	25.156	0.161
Widower (Ref)	—	—	—	—
Level of Education				
No formal education	0.175	0.065	0.47	0.001*
Primary	0.427	0.205	0.888	0.023*
Secondary	0.374	0.164	0.856	0.020*
Tertiary (Ref)	—	—	—	—
Average Monthly Income				
< ₦30,000	1.976	0.952	4.101	0.067
≥ ₦30,000 (Ref)	—	—	—	—
HIV Status				
Positive	35.303	10.045	124.072	<0.001*
Negative (Ref)	—	—	—	—
Diabetes				
Yes	0.577	0.15	2.22	0.424
No (Ref)	—	—	—	—

## Discussion

The prevalence of depression among TB patients in this study was 43.8%, which is lower than other reported prevalences of 51.9%, 52.1%, and 54.0% [6-8], respectively, from studies in Ethiopia and South Africa (64.3%) [10]. It is, however, closer to the prevalence reported in Nigeria (45.5% and 48.6%) [3,4]. These differences may be due to population characteristics, prevalence among multidrug-resistant TB patients, timing of assessment, or the phase of TB treatment.

This study found that lack of formal education, sex, and marital status were statistically associated with depression at the bivariate level. Although male sex was statistically associated with depression, it was not a predictor in the multivariate analysis. This contrasts with other studies where female gender was a significant predictor, likely due to the global higher prevalence of depression among women and factors such as hormonal influences, household responsibilities, and societal roles [5,8]. No associations were found between age, religion, ethnicity, and depression in this study, though some studies report old age as a significant predictor of depression among TB patients [5,7]. Older individuals may be more vulnerable to depression due to financial difficulties, TB-related stigma, and side effects of anti-TB drugs [2]. Low socioeconomic status among drug-susceptible TB patients was also statistically associated with depression at the bivariate level, consistent with other studies [2,7]. Patients earning less than the national minimum wage were more likely to be depressed, although this was not a significant predictor in multivariate analysis.

Interestingly, unlike many studies that identify no formal education as a predictor of depression [4,6], this study found that patients without formal education had lower odds of depression. Individuals without formal education may have limited mental health literacy, which affects their ability to recognize and articulate depressive symptoms [11].

TB/HIV comorbidity and diabetes mellitus were found to be statistically associated with depression at the bivariate and multivariate levels, consistent with findings in other research [3,8]. TB-HIV co-infection may increase depression due to the stigma and psychosocial burden of HIV [7]. In this study, patients with TB-HIV co-infection were found to have higher odds of depression, a rate considerably higher than those reported in other studies [2,6,11]. However, the large adjusted odds ratio was accompanied by a wide confidence interval, indicating imprecision and the need for cautious interpretation.

Limitations of this study

The study has some potential limitations. Since this was a cross-sectional study, it cannot establish a causal relationship. Moreover, as it represents a snapshot taken over a short period, the findings might differ in a longitudinal study. Additionally, the self-reported comorbidities could introduce potential reporting bias, and the wide confidence intervals for the association between the comorbidity of HIV with TB need to be interpreted with caution.

Data availability

This article was previously posted to the medRxiv preprint server on February 27, 2025 [12].

## Conclusions

This study concluded that although the prevalence of depression among the TB patients was lower than that reported in similar studies, a significant portion of the patients had depression, and this requires attention. Factors such as no formal education and comorbidities like TB/HIV and diabetes mellitus were statistically associated with depression.

The findings underscore the need for integrated mental health screening and support specifically within TB clinics, especially for patients with TB/HIV co-infection and low socioeconomic status. It is recommended that the Ministry of Health, in collaboration with local health authorities and TB control programs, implement routine mental health assessments and provide targeted psychosocial interventions to reduce the burden of depression in this vulnerable group.

## Appendices

This questionnaire will assess you for depression, and we can assure you that any information provided here will be kept confidential. Your data will be used anonymously, and the summary of the results will be published in a medical journal.

Kindly note that participation in this study is voluntary and will not be used against you in any way.

If you CONSENT to give your information to be used in this study, kindly append your signature below.

Signature: …………………

LGA TB No: …………………

Section A: Background status

1. Age (as at last birthday): …….

2. Sex: a) Male ☐ b) Female ☐

3. Religion: a) Christian ☐ b) Muslim ☐ c) Traditional ☐ d) Others ☐

4. Marital status: a) Single ☐ b) Married ☐ c) Widow ☐ d) Widower ☐

5. Ethnicity: a) Yoruba ☐ b) Igbo ☐ c) Hausa ☐ d) Others; specify………...

6. Level of education: a) No education ☐ b) Primary education ☐ c) Secondary ☐ d) tertiary ☐

7. Occupational status: a) Employed ☐ b) Unemployed ☐ c) Student ☐

8. Average income per month a) Less than ₦30 000 ☐ b) Above ₦30 000 ☐

9. What regimen of treatment are you on? a) Regimen 1 ☐ b) Regimen 2 ☐

10. What phase of treatment are you in? a) Intensive phase ☐ b) Continuation phase ☐

Section B: Lifestyle and other comorbid conditions among respondents

11. Do you drink alcohol? a) Yes ☐ b) No ☐

12. If yes, how many bottles per day? ….…

13. Do you smoke? a) Yes ☐ b) No ☐

14. If yes, how many sticks per day? ……

15. Body mass index

Weight: ------------------- (kg)

Height: -------------------- (m)

16. Do you have diabetes? a) Yes ☐ b) No ☐ c) Not sure ☐

17. Do you have hypertension? a) Yes ☐ b) No ☐ c) Not sure ☐

18. HIV status: a) HIV positive ☐ b) HIV negative ☐ (Confirm from patient treatment card)

19. Any previous history of mental illness(es)? a) Yes ☐ b) No ☐

20. Any family history of mental illness(es)? a) Yes ☐ b) No ☐

## References

[1] Duko B, Bedaso A, Ayano G (2020). The prevalence of depression among patients with tuberculosis: a systematic review and meta-analysis. Ann Gen Psychiatry.

[2] Yohannes K, Mokona H, Abebe L, Feyisso M, Tesfaye A, Tesfaw G, Ayano G (2020). Prevalence of depressive symptoms and associated factors among patients with tuberculosis attending public health institutions in Gede'o zone, South Ethiopia. BMC Public Health.

[3] Adebisi YA, Agumage I, Sylvanus TD (2019). Burden of tuberculosis and challenges facing its eradication in West Africa. Int J Infect.

[4] Amole TG, Yusuf AH, Salihu AS, Tsiga-Ahmed FI (2020). Prevalence and predictors of depression among tuberculosis patients in Kano, North-West Nigeria. Niger J Med.

[5] Ige OM, Lasebikan VO (2011). Prevalence of depression in tuberculosis patients in comparison with non-tuberculosis family contacts visiting the DOTS clinic in a Nigerian tertiary care hospital and its correlation with disease pattern. Ment Health Fam Med.

[6] Dasa TT, Roba AA, Weldegebreal F (2019). Prevalence and associated factors of depression among tuberculosis patients in Eastern Ethiopia. BMC Psychiatry.

[7] Abdurahman S, Yadeta TA, Ayana DA, Kure MA, Ahmed J, Mehadi A (2022). Magnitude of depression and associated factors among patients on tuberculosis treatment at public health facilities in Harari regional state, Eastern Ethiopia: multi-center cross-sectional study. Neuropsychiatr Dis Treat.

[8] Ambaw F, Mayston R, Hanlon C, Alem A (2017). Burden and presentation of depression among newly diagnosed individuals with TB in primary care settings in Ethiopia. BMC Psychiatry.

[9] Kroenke K, Spitzer RL, Williams JB (2001). The PHQ-9: validity of a brief depression severity measure. J Gen Intern Med.

[10] Thungana Y, Wilkinson R, Zingela Z (2022). Comorbidity of mental ill-health in tuberculosis patients under treatment in a rural province of South Africa: a cross-sectional survey. BMJ Open.

[11] Salnikova A, Makarenko O, Sereda Y, Kiriazova T, Lunze K, DeHovitz J, Ompad DC (2025). Depression among people living with tuberculosis and tuberculosis/HIV coinfection in Ukraine: a cross-sectional study. Glob Health Action.

[12] Olarewaju SO, Adeyemo SC, Kayode AT (2025). Prevalence, pattern, and correlates of depression among drug-susceptible tuberculosis patient enrollees in Ogbomoso, Oyo State: a cross-sectional study [IN PRESS]. medRXiv.

